# Comparison of superior vena cava and femoroiliac vein pressure according to intra-abdominal pressure

**DOI:** 10.1186/2110-5820-2-21

**Published:** 2012-06-28

**Authors:** Hafid Ait-Oufella, Pierre-Yves Boelle, Arnaud Galbois, Jean-luc Baudel, Dimitri Margetis, Mikael Alves, Georges Offenstadt, Eric Maury, Bertrand Guidet

**Affiliations:** 1 AP-HP, Hôpital Saint-Antoine, Service de réanimation médicale, Paris, 75571 Cedex 12, France; 2 Inserm U970, Paris Research Cardiovascular Center, Paris, France; 3 AP-HP, Hôpital Saint-Antoine, Service de santé publique, Paris, F-75012, France; 4 Université Pierre et Marie Curie-Paris 6, Paris, France; 5 Inserm U707, Paris, F-75012, France; 6 Service de Réanimation médicale, Hôpital Saint-Antoine, 184 rue du Faubourg Saint-Antoine, Paris, 75571 cedex 12, France

**Keywords:** Intensive unit care, Central venous pressure, Superior vena cava, Femoroiliac vena, Intra-abdominal pressure

## Abstract

**Background:**

Previous studies have shown a good agreement between central venous pressure (CVP) measurements from catheters placed in superior vena cava and catheters placed in the abdominal cava/common iliac vein. However, the influence of intra-abdominal pressure on such measurements remains unknown.

**Methods:**

We conducted a prospective, observational study in a tertiary teaching hospital. We enrolled patients who had indwelling catheters in both superior vena cava (double lumen catheter) and femoroiliac veins (dialysis catheter) and into the bladder. Pressures were measured from all the sites, CVP, femoroiliac venous pressure (FIVP), and intra-abdominal pressure.

**Results:**

A total of 30 patients were enrolled (age 62 ± 14 years; SAPS II 62 (52–76)). Fifty complete sets of measurements were performed. All of the studied patients were mechanically ventilated (PEP 3 cmH_2_0 (2–5)). We observed that the concordance between CVP and FIVP decreased when intra-abdominal pressure increased. We identified 14 mmHg as the best intra-abdominal pressure cutoff, and we found that CVP and FIVP were significantly more in agreement below this threshold than above (94% versus 50%, *P* = 0.002).

**Conclusions:**

We reported that intra-abdominal pressure affected agreement between CVP measurements from catheter placed in superior vena cava and catheters placed in the femoroiliac vein. Agreement was excellent when intra-abdominal pressure was below 14 mmHg.

## Background

Central venous pressure (CVP) is a hemodynamic parameter required in critically ill patients [1]. Conventionally, venous access is preferred via the internal jugular or subclavian vein with measurements of CVP in the superior vena cava above the right atrium. However, this approach is potentially hazardous, risking carotid artery puncture, pneumothorax, and neurologic damage [2,3]. Central venous access also may be achieved via the femoral vein by positioning the catheter in the abdominal cava/common iliac vein or in the right atrium depending of the length of the catheter. Nahum et al. reported a good correlation and agreement between CVP measurements from catheter placed in the right atrium and catheters placed in the abdominal cava/common iliac vein in children [4]. Joynt’s group confirmed these data in adults. They showed an equally good agreement between measurement of CVP in the superior vena cava above the right atrium and measurements recorded from long (40–70 cm) and short (15–20 cm) catheters placed in the inferior vena cava, respectively, close to right atrium [5] and in the common iliac vein [6]. Moreover, the mode of mechanical ventilation did not affect overall agreement between measurements [7].

In the intensive unit care (ICU), various clinical conditions, such as post-abdominal surgery, trauma, sepsis, pancreatic, and burns can lead to an increase of the intra-abdominal pressure [8]. The worst threatening condition, called abdominal compartment syndrome, alters global hemodynamic status, organ perfusion mainly through a decrease of venous return [9] and can cause death. We hypothesized that intra-abdominal hypertension could alter measurement of venous pressure and modify agreement between measurements from catheter placed in the superior vena cava, reflecting CVP, and catheter placed in the femoroiliac vein. We conducted a prospective, observational study to compare pressure measured in the superior vena cava (CVP) and in the femoroiliac vein (FIVP) according to intra-abdominal pressure.

## Methods

This study was classified as observational by the institutional review board of our hospital (Comité de Protection des Personnes de l’Hôpital Saint-Antoine). After obtaining oral consent from the patients or from their nearest relatives, we included consecutive patients who had indwelling catheters in both the superior vena cava and femoroiliac veins. Patients were under mechanical ventilation (assisted control ventilation mode) and adapted to the ventilator. Standard double-lumen catheters and renal dialysis catheters (20-cm long) were all used in this study to measure respectively superior vena cava pressure (CVP) and femoroiliac venous pressure (FIVP). All catheters used in this study were already in place and justified by the patient’s clinical condition; no catheter was specifically inserted for the sole purpose of the study. Patients could be included twice if the second set of measurements was performed more than 7 days after the first inclusion. Venous pressure measurements were recorded at both sites by using the same pressure transducer. Before the measurements, a zero pressure calibration during the tele-expiratory period was performed on each catheter, with the patient in a supine position. All pressure measurements (intra-abdominal and venous) were repeated four times at 5-min intervals during the tele-expiratory period, and mean values were calculated.

Intra-abdominal pressure was measured indirectly by measuring the pressure within the bladder, as described by Cheatham et al. [10]. Briefly, two, three-way stopcocks are connected serially to a disposable pressure transducer. A standard intravenous infusion set is connected to 1 L of sterile normal saline and attached to one stopcock, and a 20-mL syringe is attached to the second stopcock. An 18-gauge angiocatheter is inserted into the culture aspiration port of the urinary drainage tube using aseptic technique. The needle is removed, leaving the plastic infusion catheter in place. The infusion catheter is attached to the first stopcock via pressure tubing. The system is flushed with normal saline and the pressure transducer “zeroed” at the level of the symphysis pubis. To measure intra-abdominal pressure, the urinary drainage tube is clamped immediately distal to the catheter. The stopcocks are turned off to the patient and to the pressure transducer. Normal saline is aspirated from the intravenous bag using the 20-mL syringe. The first stopcock is turned on to the patient, and the normal saline (20 ml) is instilled into the bladder through the urinary catheter [11]. The stopcocks are then turned off to the syringe and intravenous tubing. The clamp on the urinary drainage tubing is momentarily released to ensure that all air is flushed from the urinary catheter. The patient’s intra-abdominal pressure is then measured at end-expiration. The clamp is removed and the bladder allowed to drain.

Exclusion criteria were mechanical ventilation mode different than ACV and agitation, and disadaptation to the ventilator that troubled superior vena cava pressure measurements.

### Statistical analysis

Data were expressed as mean ± standard deviation or median (interquartile range) in the case of non-Gaussian distribution. The intraclass correlation coefficient (ICC) was used to assess concordance between FIVP and CVP. ICC values >0.8 correspond to an “excellent” agreement (Landis and Koch). To assess the impact of intra-abdominal pressure, the ICC was calculated for subsets of patients with less than a given intra-abdominal pressure threshold. The threshold value was systematically varied from x to y to find the best intra-abdominal pressure cutoff; x was the lower intra-abdominal pressure obtained in this study and y the highest. After identification of the best intra-abdominal pressure cutoff, the ICC was compared for observations below and above the threshold using a bootstrap-based test. Finally, in observations where the intra-abdominal pressure was less than the optimal cutoff, a linear regression model was used to predict CVP from FIVP.

We hypothesized that agreement would be very good in practice (ICC ~90%) when intra-abdominal pressure was low. Including 15 patients was sufficient to get a lower 95% confidence interval (CI) >70%. Assuming that the prevalence of large IAP could be up to 50%, we finally included 30 patients. All analyses were made by using R software (v 2.9.1; http://cran.r-project.org).

## Results

Between February 2009 and August 2010, 30 consecutive patients were enrolled, and 50 complete sets of measurements were performed (Table 1). All patients had a subclavicular or jugular catheter for drug infusion and a 20-cm long, femoral catheter for renal dialysis. The position of catheter in the superior vena cava was confirmed by radiography, within 5 cm to the junction vena cava-right atrium. We observed during a preliminary study using abdominal CT scan that 20-cm long, femoral catheters were placed at the junction iliac vein/inferior vena cava. All of the studied patients were mechanically ventilated and had acute renal failure requiring dialysis. In the group with a PIA ≥14 mmHg, patients had mostly intra-abdominal infection or abdominal surgery, whereas in the group of patients with a PIA <14 mmHg, admission diagnoses were various, such as lung infection and cardiac arrest (Table 2). Among patients admitted for septic shock, the proportion of cirrhosis with ascites was more important in the group with high IAP (5/8 versus 3/12 patients). Severity was high as illustrated by high SAPS II.

**Table 1:** Characteristic of included patients

	
Patients (n)	30
Weight (kg)	80 ± 16
BMI (kg/m^2^)	29.8 ± 6.3
Gender (M/F)	15/15
Age (yr)	62 ± 14
SAPS II	62 (52–76)
Dialysis, n (%)	30 (100)
Vasopressors, n (%)	27 (90)
Plateau pressure (cmH_2_0)	20 (18–25)
PEEP (cm H_2_0)	3 (2–5)

**Table 2:** Causes of admission according to the intra-abdominal pressure (IAP)

	
PIA <14 mmHg	PIA ≥14 mmHg
Septic shock	Septic shock
Lung (7)	Lung (2)
Abdomen (4)	Abdomen (4)
Soft tissue (1)	Urinary tract (1)
Urinary tractus (1)	Unlocalized bacteriemia (1)
Endocarditis (1)	
Unlocalized bacteriemia (1)	Liver transplantation (2)
	Acute alcoholic hepatitis (2)
Acute alcoholic hepatitis (2)	
Cardiac arrest (4)	Gut occlusion (1)
Cardiogenic shock (3)	Mesenteric infarction (1)
HELP syndrome (2)	
Self poisoning (2)	
Other (8)	

**Figure 1:**
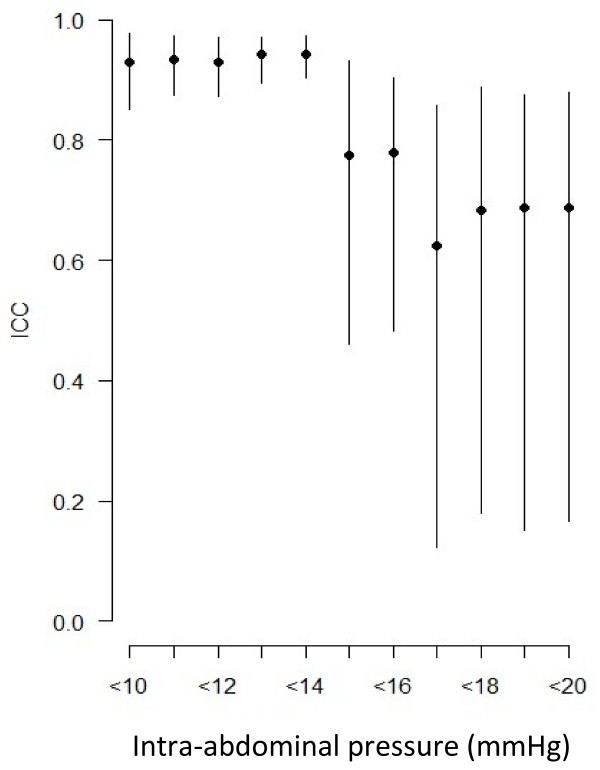
**Intraclass correlation coefficient (ICC) for patients with intra-abdominal pressure less than a given value (dots for ICC values and lines for 95% confidence interval).** When patients with high intra-abdominal pressure were added, ICC decreased.

**Figure 2:**
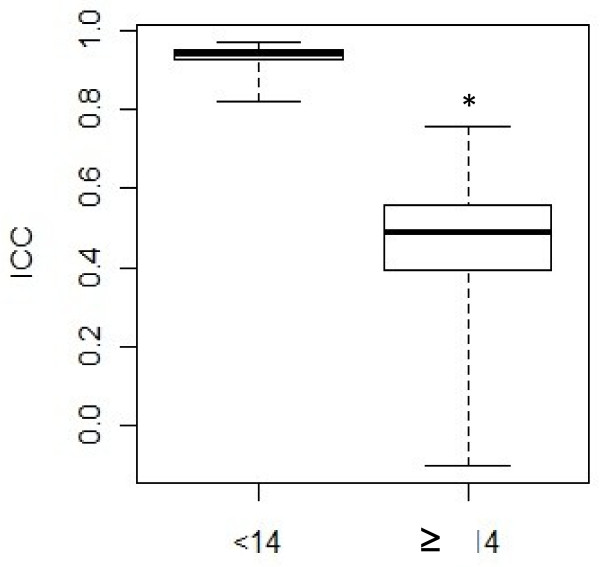
**Intraclass correlation coefficient according to intra-abdominal pressure cutoff.** Less than 14 mmHg, agreement between FIVP and CVP measurements was excellent and better than situations when intra-abdominal pressure was >14 mmHg (0.94 versus 0.5, *P* = 0.002). FIVP, femoroiliac venous pressure; CVP, central venous pressure. Boxes show first and third quartiles, with the median as a thick line. Whiskers extend to 1.5 interquartile range (Q75-Q25).

Interestingly, we observed that the concordance between CVP and FIVP measurements decreased when intra-abdominal pressure increased (Figure 1). The ICC was 0.94 (95% CI [0.89-0.97]), corresponding to excellent agreement on the Landis and Koch scale, when considering patients with intra-abdominal pressure <14 mmHg. The ICC reduced to 0.77 (95% CI [0.41-0.94]) when patients with intra-abdominal pressure <15 mmHg were added, and concordance decreased even more as patients with larger intra-abdominal pressure values were considered (Figure 1). Therefore, concordance between CVP and FIVP was best when IAP was <14 mmHg. CVP and FIVP were significantly more in agreement below this threshold than above (0.94 versus 0.5, *P* = 0.002; Figure 2).

**Figure 3:**
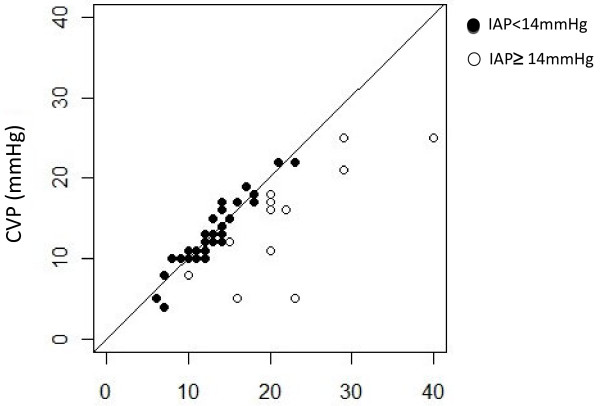
**Less than 14 mmHg (black circle) FIVP reflected CVP, the linear regression equation was CVP = 1.01 FIVP. Greater than 14 mmHg (white circle), FIVP overestimated CVP.** FIVP, femoroiliac venous pressure; CVP, central venous pressure.

As expected from the large concordance, CVP was very well predicted by FIVP in patients with intra-abdominal pressure <14 mmHg. The linear regression equation was CVP = 1.01 FIVP (*R*^*2*^ = 0.99; Figure 3).

## Discussion

Our results showed that the FIVP could be used to estimate correctly CVP and confirmed previous published studies in adults and children in ICU [4,5]. However, we pointed out that FIVP measurement accuracy had to be interpreted according to the intra-abdominal pressure. When intra-abdominal pressure was inferior to 14 mmHg, FIVP predicted very well CVP. When intra-abdominal pressure was more than 14 mmHg, corresponding to the first stage of intra-abdominal hypertension, FIVP did not reflect accurately CVP anymore. The difference could be large (median 4 (range 0–18)) and was always in the direction FIVP > CVP. Interestingly, this cutoff was close to the threshold (12 mmHg) that defines intra-abdominal hypertension according to the *International Conference of Experts on Intra-abdominal Hypertension and Abdominal Compartment Syndrome*[12]*.* It is not exactly the same value of cutoff probably because the studied population size was limited. In case of severe intra-abdominal hypertension (IAP > 20 mmHg), De Keulenaer et al. [13] recently reported that FIVP could be used as a surrogate measure of IAP, illustrating the link between FIVP and IAP. The consequences during severe infections, if physicians followed *Survival Sepsis Campaign Guidelines* for fluid administration, could be an underestimation of hypovolemia in these patients, a delay for fluid challenge and could *in fine* worsen patient’s prognosis [14]. The effects of intra-abdominal pressure on FIVP measurements are relevant, because abdominal compartment syndrome is not rare in ICU (from 1–20% according to published data) and is frequently underestimated [15].

More generally, this study questions about the effects of intra-abdominal pressure on others hemodynamic tools used in the clinical setting to evaluate volemia. For example, passive leg raising was reported by several groups to be a good predictor of fluid responsiveness [16]. However, Mahjoub et al. showed that the passive left raising maneuver did not accurately predict fluid responsiveness in patients with intra-abdominal hypertension [17].

Our study has several limitations. It is a monocentric study and results have to be confirmed in a larger population. Nevertheless, although the size of this preliminary study was not very large, it was sufficient to highlight significant results. Intra-abdominal pressure was recorded indirectly by using intra-bladder pressure, but this technique had achieved a widespread adoption worldwide. Finally, our studied population did not have severe pulmonary disease: median PEEP was 3 cmH_2_O (2–5) and median plateau pressure was 20 cmH_2_O (18–25). Therefore, our data could not be extrapolated to clinical situations with lung injury and/or high PEEP and/or high intrathoracic pressure, but we could speculate that, in these situations, agreement between central and femoroiliac venous pressure would be strongly altered. The impact of increased intra-abdominal pressure on agreement between CVP and FIVP was documented using short catheters (20-cm long) and could not be extrapolated to measurements obtained using longer catheters, which tip arise right atrium.

## Conclusions

We reported the effects of intra-abdominal pressure on venous pressure measurements. When the intra-abdominal pressure was inferior to 14 mmHg, femoroiliac pressure predicted very well central venous pressure. However, when intra-abdominal pressure is >14 mmHg, femoroiliac pressure did not and intra-class correlation coefficient decreased from 94% to 50%.

## Competing interest

The authors had no conflict of interest.

## Authors’ contributions

HAO, GO, EM and BG designed the study. HAO and PYB performed the statistical analysis. HAO, AG, JLB, DM and MA participated to patients’ inclusion. All authors read and approved the final manuscript.
